# The RNF/NQR redox pumps: a versatile system for energy transduction in bacteria and archaea

**DOI:** 10.1007/s00253-025-13531-0

**Published:** 2025-06-17

**Authors:** Wolfgang Buckel, Ulrich Ermler, Janet Vonck, Günter Fritz, Julia Steuber

**Affiliations:** 1 Faculty of Biology, Philipps-Universität Marburg, Karl-von-Frisch-Straße 8, 35043 Marburg, Germany; 2 Department of Molecular Membrane Biology, Max Planck Institute of Biophysics, Max-von-Laue-Straße 3, 60438 Frankfurt am Main, Germany; 3 Department of Structural Biology, Max Planck Institute of Biophysics, Max-von- Laue-Straße 3, 60438 Frankfurt am Main, Germany; 4 Department of Cellular Microbiology, Institute of Biology, University of Hohenheim, Garbenstraße 30, 70599 Stuttgart, Germany

**Keywords:** Electron transport, Respiration, Electrochemical proton gradient, Electrochemical sodium gradient, RNF, NQR

## Abstract

**Abstract:**

The Na^+^ (or H^+^)-translocating ferredoxin:NAD^+^ oxidoreductase (also called RNF, rhodobacter nitrogen fixation, complex) catalyzes the oxidation of reduced ferredoxin with NAD^+^, hereby generating an electrochemical gradient. In the reverse reaction driven by an electrochemical gradient, RNF provides reduced ferredoxin using NADH as electron donor. RNF plays a crucial role in the metabolism of many anaerobes, such as amino acid fermenters, acetogens, or aceticlastic methanogens. The Na^+^-translocating NADH:quinone oxidoreductase (NQR), which has evolved from an RNF, is found in selected bacterial groups including anaerobic, marine, or pathogenic organisms. Since NQR and RNF are not related to eukaryotic respiratory complex I (NADH:quinone oxidoreductase), members of this oxidoreductase family are promising targets for novel antibiotics. RNF and NQR share a membrane-bound core complex consisting of four subunits, which represent an essential functional module for redox-driven cation transport. Several recent 3D structures of RNF and NQR in different states put forward conformational coupling of electron transfer and Na^+^ translocation reaction steps. Based on this common principle, putative reaction mechanisms of RNF and NQR redox pumps are compared.

**Key points:**

• *Electrogenic ferredoxin:NAD*^+^ *oxidoreductases (RNF complexes) are found in bacteria and archaea.*

• *The Na*^+^ *-translocating NADH:quinone oxidoreductase (NQR) is evolutionary related to RNF.*

• *The mechanism of energy conversion by RNF/NQR complexes is based on conformational coupling of electron transfer and cation transport reactions.*

**Supplementary Information:**

The online version contains supplementary material available at 10.1007/s00253-025-13531-0.

## Introduction

In most chemotrophic microorganisms, the endergonic synthesis of ATP is inevitably linked to the oxidation of a substrate and reduction of an acceptor molecule. The continuous re-oxidation of this acceptor is crucial to maintain ATP synthesis and ultimately requires excretion of fermentation products as in substrate level phosphorylation, or utilization of exogenous electron acceptors as in oxidative phosphorylation. In both scenarios, cellular electron carriers such as NADH/NAD^+^ and reduced/oxidized ferredoxin (Fd) play an important role. Both carriers act as substrates for the membrane-bound RNF complex (rhodobacter nitrogen fixation complex, also termed NFO, NAD^+^:ferredoxin oxidoreductase complex). The name RNF was based on the finding that the six genes *rnfABCDE* were essential for N_2_ fixation in *Rhodobacter capsulatus* (Schmehl et al. 1993). The authors proposed that these genes encode subunits of a membrane-bound electron transfer complex, which provides low-potential electrons in the form of reduced ferredoxin for the reduction of N_2_ by nitrogenase. This function of RNF was also confirmed in other N_2_ fixing bacteria such as *Azotobacter vinelandii* (Martin Del Campo et al. 2022). Here, RNF exploits the proton motive force (pmf) of the bacterial membrane to push electrons from NADH to the lower-potential ferredoxin, operating in the so-called *reverse electron transfer* mode. RNF complexes from amino acid-fermenting bacteria (Brüggemann et al. 2003; Boiangiu et al. 2005; Vitt et al. 2022) and the related Na^+^-translocating NADH:quinone oxidoreductase (NQR) (Hau et al. 2023) preferably operate in the so-called *forward electron transfer* mode, coupling the electron transfer from a low-potential to a high-potential substrate to the transport of Na^+^ from the cytoplasm to the periplasm under formation of an electrochemical Na^+^ gradient (ΔµNa^+^). Depending on the growth substrates, the RNF in acetogenic bacteria is crucial for establishing ΔµNa^+^ (*forward electron transfer mode*), or for the formation of reduced ferredoxin from NADH driven by ΔµNa^+^ (*reverse electron transfer mode*) (Schuchmann and Müller 2016; Westphal et al. 2018). RNF represents an important metabolic oxidoreductase as it links the redox reactions between NADH/NAD^+^ and ferredoxin_ox/red_ to the formation or utilization of electrochemical Na^+^ or H^+^ gradients. In these different roles, it is widespread in the bacterial kingdom and also present in certain archaeal lineages (Hess et al. 2016). In marked contrast to RNF, the NADH:quinone oxidoreductase (NQR) is found only in some bacterial groups. An ancient RNF represents the ancestor of NQR, which first appeared in the Bacteroidetes (Munoz et al. 2020). A comparison of the six Nqr subunits with the six Rnf subunits reveals a conserved core of four membrane-bound subunits (RnfD/NqrB, RnfG/NqrC, RnfE/NqrD, RnfA/NqrE) (Table 1). This suggests that the mechanism of redox-driven cation translocation across the membrane is shared between RNF and NQR. In RNF and the related NQR, we find both Na^+^ and H^+^ dependent complexes.

**Table 1:** Comparison of subunits of RNF and NQR complexes

NQR *V. cholerae*	RNF *C. tetanomorphum*	RNF1 *A. vinelandii*	RNF *A. woodii*
Hau et al. (2023)	Vitt et al. (2022)	Zhang and Einsle (2024)	Kumar et al. (2025)
PDB code 8 A1U	PDB code 7ZC6	PDB code 8 AHX	PDB code 9ERK
	Subunit	Subunit	Subunit
	Cofactors	Cofactors	Cofactors
	RMSD (to corresponding NQR subunit)	RMSD (to corresponding NQR subunit)	RMSD (to corresponding NQR subunit)
	Sequence identity (with corresponding NQR subunit)	Sequence identity (with corresponding NQR subunit)	Sequence identity (with corresponding NQR subunit)
NqrA	RnfC	RnfC	RnfC
No cofactor	1 FMN	1 FMN	1 FMN
	2 [4Fe-4S]	2 [4Fe-4S]	2 [4Fe-4S]
	4.05 Å (403 C,α 93% of all)	4.02 Å (404 Cα, 85% of all)	4.14 Å (404 Cα, 91% of all)
	17.7% (403 residues)	13.9% (404 residues)	15.6% (404 residues)
NqrB	RnfD	RnfD	RnfD
1 riboflavin	1 riboflavin	1 riboflavin	1 riboflavin
1 covalent FMN	1 covalent FMN	1 covalent FMN	1 covalent FMN
1 ubiquinone	1.47 Å (271 Cα, 89% of all)	1.46Å (288 C 83% of all)	1.38 Å (275 C 86% of all)
	35.0% (271 residues)	28.8% (288 residues)	34.2% (275 residues)
NqrC	RnfG	RnfG	RnfG
1 covalent FMN	1 covalent FMN	1 covalent FMN	1 covalent FMN
	2.82 Å (183 Cα, 98% of all)	3.33 Å (191 Cα, 97% of all)	3.26 Å (190 C, 92% of all)
	19.1% (183 residues)	16.2% (191 residues)	16.8% (190 residues)
NqrD	RnfE	RnfE	RnfE
1 [2Fe-2S] (between NqrD/E)	1 [2Fe-2S] (between RnfA/E)	1 [2Fe-2S] (between RnfA/E)	1 [2Fe-2S] (between RnfA/E)
	1.48 Å (190 Cα, 98% of all)	1.89 Å (195 Caα, 91% of all)	1.57 Å (193 Cα, 98% of all)
	37.9% (190 residues)	34.9% (195 residues)	37.8% (193 residues)
NqrE	RnfA	RnfA	RnfA
	2.16 Å (187 Cα, 98% of all)	1.16 Å (189 Cα, 100% of all)	2.05 Å (188 Cα, 98% of all)
	39.0% (187 residues)	38.6% (189 residues)	43.1% (193 residues)
-	RnfB	RnfB	RnfB
	5 [4Fe-4S]	2 [4Fe-4S]	7 [4Fe-4S]
	1 [4Fe-4S] in flexible domain	1 [4Fe-4S] in flexible domain	1 [4Fe-4S] in flexible domain
NqrF	-	-	-
1 FAD			
1 [2 Fe-2S]			
1 NADH			
-	-	RnfH	-
		No cofactor	

No homologous subunit in other complexes

Structural alignments were prepared with TM-align (Zhang 2005)

Ultimately, the question is whether ancient ATP synthases were driven by an electrochemical Na^+^ or proton gradient. Studying the evolution of F-type versus V-type ATP synthases and considering Na^+^ or H^+^ specificities of the enzymes as predicted from sequence comparisons, Galperin and colleagues argue that Na^+^-specific ATP synthases predated H^+^-dependent enzymes and propose that ancestral membrane bioenergetics used Na^+^ (Mulkidjanian et al. 2008). This is in contrast to a hydrothermal vent scenario of the origin of life where the energy metabolism of early cells is powered by existing pH gradients (outside acidic) (Lane 2020), and primordial, energy-converting protein complexes are proposed to be proton-dependent. While the question of cation specificity of primordial pumps is not easily settled, it seems very plausible that an ATP synthase operating together with RNF represents an ancient chemiosmotic system, which still prevails in the metabolism of many bacteria and archaea (Marreiros et al. 2016). In an elegant series of experiments using the purified complexes from *Thermotoga maritima* co-reconstituted in proteoliposomes, it was shown that the electrochemical Na^+^ gradient established by RNF drives synthesis of ATP by the Na^+^-dependent F_1_F_O_ ATP synthase (Kuhns et al. 2020b).

Here we describe the functions of selected RNF and NQR complexes, considering their role in metabolism and energy conservation in selected microorganisms. Recent findings on the structure and function of RNF and NQR are summarized. A comparative, structural analysis of both complexes reveals a highly conserved, membrane-bound core complex composed of four subunits, which interacts with different electron input and electron output modules. Considering both structural and functional studies, conformational coupling of electron transfer and cation transport processes is identified as the common mechanistic principle of the RNF/NQR oxidoreductase family.

## Physiological functions of selected RNF and NQR complexes

### RNF of *Clostridium tetani* and *C. tetanomorphum*: energy conservation during the fermentation of glutamate

The genome analysis of *C. tetani* revealed that this pathogenic bacterium possesses an RNF complex (Brüggemann et al. 2003). The related bacterium *C. tetanomorphum* has a rather high content of RNF (Boiangiu et al. 2005), which facilitated purification and subsequent structural analysis of the complex (Vitt et al. 2022). *C. tetani* and *C. tetanomorphum* ferment glutamate via (*S*,*S*)−3-methylaspartate according to the following Eq. (1):

1 $$5\;Glutamate^-\;+\;6\;H_2O\;+\;2\;H^+\;\rightarrow\;5\;NH_4^+\;+\;5\;CO_2\;+\;6\;acetate^-\;+\;2\;butyrate^-\;+\;H_2$$

(ΔG°′ = − 314 kJ).

The proposed pathway starts with the conversion of 5 (*S*)-glutamate to 5 ammonia, 5 acetate, and 5 pyruvate (Buckel and Thauer 2013). The pyruvates are oxidized to 5 CO_2_ and 5 acetyl-CoA, whereby 10 ferredoxins are reduced. From 3 acetyl-CoA, 3 ATP are synthesized via substrate level phosphorylation. Additional 4 ferredoxins are reduced via electron bifurcation during butyrate synthesis from 2 acetyl-CoA, 2 acetate, and 6 NADH. H_2_ production requires 2 reduced ferredoxins. In total, 12 reduced ferredoxins are formed serving as electron donors for RNF, which reduces 6 NAD^+^ to generate 6 NADH under translocation of 12 Na^+^. Since 5 Na^+^ are probably required for the uptake of 5 glutamates, 7 Na^+^ remain for the chemiosmotic synthesis of about 1.75 ATP. Together with the 3 ATP obtained from acetyl-CoA, 4.75 ATP are obtained from 5 glutamate (approximately 1 ATP/glutamate); see also (Buckel 2021).

### RNF of *Acetobacterium woodii*: energy conservation during reduction of CO_2_ to acetate

RNF represents an important oxidoreductase for energy conservation in *A. woodii*, which reversibly couples the oxidation of reduced ferredoxin and reduction of NAD^+^ to the translocation of Na^+^ (Hess et al. 2013; Schuchmann and Müller 2014, 2016; Westphal et al. 2018). Recently, the structure of *A. woodii* RNF was reported (Kumar et al. 2025). *A. woodii* synthesizes acetate from H_2_ and CO_2_ using the Wood-Ljungdahl pathway (Ragsdale and Pierce 2008) according to the following Eq. (2):

2 $$2\;CO_2\;+\;4\;H_2\;\rightarrow\;CH_3COOH\;+\;2\;H_2O;\;\Delta G^{\circ,}=\;-\;96\;kJ/mol\;acetate$$

The first step of the Wood-Ljungdahl pathway is the production of formate catalyzed by the hydrogen dependent CO_2_ reductase (Eq. 3):

3 $$CO_2\:+\;H_2\:\rightarrow\;HCOO^-+\:H^+$$

For the next steps reduced ferredoxin (Fdˉ) and NADH are required, which are provided by a bifurcating hydrogenase (Schuchmann and Müller 2012) (Eq. 4). This oxidoreductase catalyzes two one-electron transfers from H_2_ (*E*°′ = − 414 mV), one to the high potential NAD^+^ (*E*°′ = − 320 mV) which drives the other electron to the low potential ferredoxin (Fd, *E*°′ = − 420 to − 450 mV).

4 $$2\;H_2+\;2\;Fd\;+\:NAD^+\;\:\rightarrow\;2\;Fd^-\;+\:NADH\;+\:3\;H^+$$

The carbon metabolism proceeds by formylation of tetrahydrofolate (THF) to N^5^-formyl-THF, which requires ATP hydrolysis. The product cyclizes to N^5^-N^10^-methenyl-THF, which is reduced with 2 NADH via N^5^,N^10^-methylene-THF to N^10^-methyl-THF. The methyl group is transferred to a corrinoid-Fe-S protein and combined with CO and CoASH to afford acetyl-CoA, from which acetate and ATP are synthesized. CO is obtained by reduction of CO_2_ with 2 Fdˉ. The biochemistry of acetogenesis is reviewed in (Ragsdale 2008). The overall reaction is summarized in Eq. (5):

5 $$2\;CO_2\:+\;3\;Fd^-\;+\:2.5\;NADH\;+\:4.5\;H^+\;\:\rightarrow\;CH_3COO^-\;+\:3\;Fd\;+\:2.5\;NAD^+\;\:+\;2\;H_2O$$

The bifurcating hydrogenase reduces 4 Fd and 2 NAD^+^ with 4 H_2_. 3 Fdˉ are consumed during acetate synthesis, a fourth Fdˉ reduces 0.5 NAD^+^ catalyzed by RNF whereby 1 Na^+^ is transported across the membrane and then used for the chemiosmotic synthesis of about 0.3–0.4 ATP. Since one ATP is consumed by formylation of THF and one ATP is generated via acetyl-CoA, ΔµNa^+^ produced by RNF is crucial for energy conservation (Schuchmann and Müller 2014).

### RNF of *Azotobacter vinelandii*: the power supply for biological nitrogen fixation

Biological nitrogen fixation performed by diazotrophic bacteria requires large amounts of energy in the form of 16 ATP and 8 low potential electrons to overcome the high activation barrier for cleavage of the dinitrogen triple bond by the enzyme nitrogenase (Einsle 2023). Nitrogenase of the model diazotroph *A. vinelandii* operates at a redox potential of − 0.62 V, thus the redox potential of NADH is not sufficiently low for N_2_ fixation. In *A. vinelandii*, ferredoxin or flavodoxin therefore act as electron donor. While reduced flavodoxin is provided by the electron-bifurcating Fix system (reviewed in (Barney 2020)) ferredoxin is reduced by RNF that uses the proton motive force and NADH as electron donor in a *reverse electron transfer* reaction. *A. vinelandii* contains two *rnf* gene clusters coding for RNF1 and RNF2. RNF1 is the primary electron source for nitrogenase and the *rnf*_*1*_ gene cluster is expressed under nitrogen fixating (nif) conditions (Barney and Plunkett 2022), while the *rnf*_*2*_ gene cluster is expressed independently of a nitrogen source (Curatti et al. 2005). Recently, Zhang and Einsle (2024) have reported the structure of *A. vinelandii* RNF1. Compared to other structurally characterized RNF complexes (Vitt et al. 2022; Kumar et al. 2025), the *A. vinelandii* RNF shows some differences, in particular, an additional, cytoplasmic subunit RnfH that does not contain a redox cofactor. These differences in RNF of *A. vinelandii* RNF might be crucial to promote reduction of ferredoxin in the *reverse electron transfer* mode driven by the electrochemical proton gradient (Zhang and Einsle 2024). This proton motive force is generated by aerobic respiration providing a large surplus of ATP (Alleman et al. 2021).

### RSX of *Escherichia coli*: protection against superoxide and nitric oxide

The RsxABCDGE complex (RSX) is a homologue of the RnfABCDGE complex (RNF) and is supposed to act as NAD(P)H-dependent reducing system of the oxidized form of the transcriptional regulator SoxR, as shown for *E. coli* (Lee et al. 2022). SoxR is a small [2Fe-2S] cluster containing protein that binds to DNA (Ding et al. 1996). Oxidative stress by reactive oxygen species or nitric oxide leads to the oxidation of the [2Fe-2S] cluster from the reduced form [Fe(II)-Fe(III)] to the all-ferric form [Fe(III)-Fe(III)] thereby activating SoxR. SoxR in the oxidized active state initiates the transcription of the downstream regulator *soxS*, which in turn activates the expression of the stress regulon. Among the activated genes is *rsxABCDGE*, *rseC* and interestingly *apbE*. After the oxidative stress is relieved, the SoxR is inactivated by reduction through RsxABCDGE together with RseC (Lee et al. 2022). ApbE is a flavin transferase (Bertsova et al. 2013) that catalyzes the covalent attachment of FMN to Thr residues recognizing a specific sequence motif. Such a motif is present in RsxD (homologous to RnfD or NqrB) and RsxG (homologous to RnfG or NqrC). The reduction potential of free SoxR has been determined as − 285 mV, above of that of ferredoxin (− 420 mV) and NAD^+^ (− 320 mV) (Ding et al. 1996). The question arises, why the cell uses RSX and a ΔµNa^+^/H^+^ to reduce ferredoxin and subsequently SoxR, while the potential of NADH would be sufficient to reduce SoxR.

### RNF of archaea: aceticlastic methanogenesis in *Methanosarcina acetivorans*

The *M. acetivorans* RNF comprises, besides the six subunits RnfABCDGE, an additional multiheme *c*-type cytochrome, which is expected to facilitate electron transfer to methanophenazine or extracellular electron acceptors (Gupta et al. 2024). *M. acetivorans* critically relies on this Na^+^-translocating RNF (Schlegel et al. 2012b) to provide reduced methanophenazine for the final reduction step in methanogenesis, the reduction of the heterodisulfide CoM-S–S-CoB to CoM-SH and CoB-SH catalyzed by HdrED (heterodisulfide reductase) (Suharti et al. 2014; Ferry 2020). With the help of ATP, acetate is activated to acetyl-CoA, which in the presence of tetrahydrosarcinapterin is cleaved to CO, CoA, and methyl-tetrahydrosarcinapterin. Reduced ferredoxin, generated by the action of CO with carbon monoxide dehydrogenase, acts as electron donor for the Na^+^-translocating RNF (Schlegel et al. 2012b). Methyl-tetrahydrosarcinapterin reacts with CoM-SH to tetrahydrosarcinapterin and methyl-S-CoM, whereby the energy of the methyl-transfer from N to S is used to generate an electrochemical Na^+^ gradient (Becher et al. 1992; Lienard et al. 1996). Subsequently, methyl-S-CoM and the electron donor CoB-SH are converted to methane and the heterodisulfide CoM-S–S-CoB. The chemiosmotic potential generated by RNF and other primary pumps is used to drive ATP synthesis by the A_1_A_O_ ATP synthase, which may use Na^+^ and H^+^ as coupling cation (Schlegel et al. 2012a). However, the reported stoichiometries of Na^+^ translocated by RNF are not sufficient to provide the energy required (Schlegel et al. 2012b). Further studies indicate that also energy conservation by means of electron bifurcation is important for survival of *M. acetivorans* in native environments (Prakash et al. 2019; Song et al. 2023).

### NQR of *Vibrio cholerae*: the redox Na^+^ pump of a human pathogen

The Na^+^-translocating NQR was first discovered in marine *Vibrio* species (Unemoto et al. 1977) and was later found in many bacterial groups (Reyes-Prieto et al. 2014; Munoz et al. 2020; Sampaio et al. 2022). It operates in the *forward* mode and uses NADH as electron donor and ubiquinone as electron acceptor. Like RNF, it is composed of six subunits termed NqrABCDEF. In many pathogens such as *Vibrio cholerae*, the causative agent of cholera disease, NQR contributes to the virulence of the pathogen (Dibrov et al. 2017; Steuber and Fritz 2024). While the *V. cholerae* NQR is a Na^+^ pump (Toulouse et al. 2017), NQR from *Pseudomonas aeruginosa* transports H^+^ rather than Na^+^ (Raba et al. 2018). The central role of NQR in the energy metabolism of many pathogens, in particular of multidrug resistant Gram-negative bacteria such as *Pseudomonas aeruginosa*,* Klebsiella pneumoniae*, or *Acinetobacter baumannii* defines it as a possible target for new antibacterial drugs. NQR is also found in strict anaerobes such as *Segatella bryantii*. Here, NQR reduces menaquinone to menaquinol, which acts as electron donor for the reduction of fumarate (Schleicher et al. 2021; Hau et al. 2024).

### NQR has evolved from RNF

The structural similarity of the core subunits of RNF and NQR outlined here corroborates that NQR has evolved from RNF (reviewed in (Biegel et al. 2011)). During evolution a duplication of the *rnf* operon occurred and sequence data indicate that NQR evolved originally in the Bacteroidota (Munoz et al. 2020), which radiated about 1.7 billion years ago (Ward and Shih 2022). The loss of the *rnfB* gene from this duplicated operon was presumably compensated by acquiring a gene coding for a monooxygenase reductase of the FNR family. This gene codes for NqrF in NQR and replaces RnfB as electron input module. It seems likely that NQR originally evolved as a NADH: menaquinone reductase and ubiquinone reductase activity has evolved later, suggesting that NQR reducing menaquinone like the one from *Segatella bryantii* (Schleicher et al. 2021; Hau et al. 2024) represents an evolutionary older homolog.

### Subunits, cofactors, and electron transfer in RNF/NQR complexes

A series of recent structures of Na^+^-NQR and RNF complexes yielded detailed insights into the architecture and function of these redox-driven pumps (Kishikawa et al. 2022; Vitt et al. 2022; Hau et al. 2023; Zhang and Einsle 2024; Kumar et al. 2025). A compilation of structural information available for RNF and NQR, as well as for individual NQR subunits from different bacteria is provided in the electronic supplementary material (Table S1). Functional properties of RNF and NQR which are derived from biochemical and physiological experiments are summarized in Table S2.

**Fig. 1:**
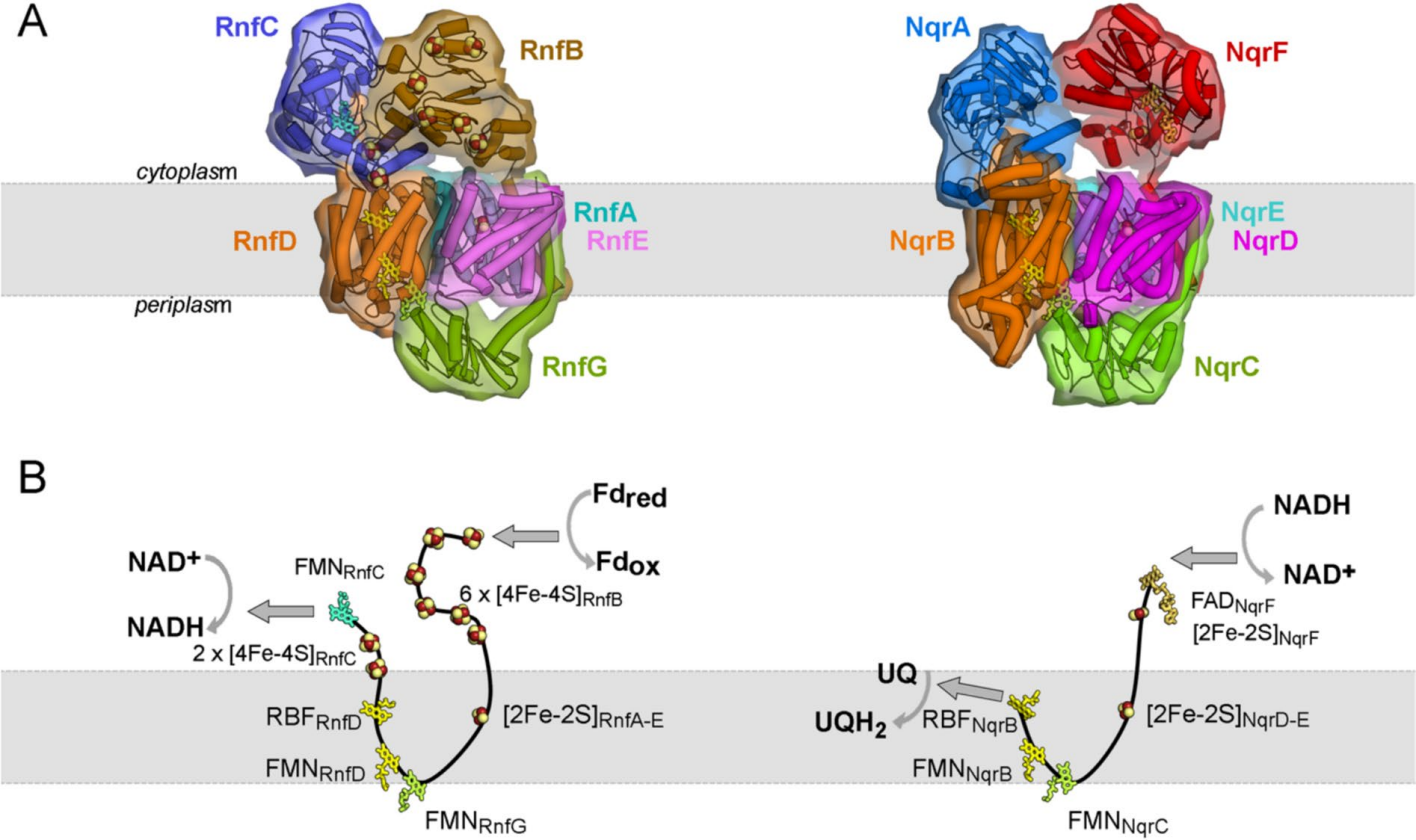
Comparison of RNF (*C. tetanomorphum*) and NQR (*V. cholerae*) architecture and electron transfer pathways. **A **Left, RNF from *C. tetanomorphum* (pdb code 7zc6); right, NQR from *V. cholerae* (pdb code 8a1w). The ferredoxin domain of RnfB, which was not resolved in the cryo-EM map, was modeled here by AlphaFold (Jumper et al. 2021) to complete the structure. Five out of the six subunits of both complexes share high homology as indicated by similar coloring. RnfC/NqrA: blue, RnfD/NqrB: orange, RnfG/NqrC: green, RnfA/NqrE: cyan, RnfE/NqrD: magenta; RnfB: brown, NqrF: red. **B **The cofactors of the integral transmembrane subunits RnfD/NqrB, RnfA/NqrE, RnfE/NqrD, and RnfG/NqrC are strictly conserved and reside at almost exactly the same positions in both complexes revealing an identical transmembrane electron-transfer pathway in RNF and NQR. RnfB subunit with six [4Fe-4S] clusters is structurally not related to NqrF, which harbors a FAD and a [2Fe-2S] cluster. RnfC and NqrA are structurally closely related; however, NqrA lacks any redox cofactors. The function of RnfC in the RNF complex is to transfer electrons from riboflavin_RnfD_ to the electron acceptor NAD^+^, while in NQR the electrons are directly transferred to ubiquinone in the membrane. The grey arrows indicate the forward reaction from a low potential electron donor to a high potential electron acceptor

Despite the different functions of RNF and NQR in the diverse organisms most of their subunits are conserved and, in particular, the four membrane-bound subunits RnfAEDG and NqrEDBC exhibit a strikingly high structural similarity (Fig. 1). These four subunits constitute the energy-conserving core machinery and harbor a set of unusual redox cofactors: an intramembranous [2Fe-2S] cluster, two covalently attached FMNs, and a riboflavin (Fig. 1). The extraordinary properties of these cofactors are emphasized by the necessity to deploy additional proteins for the insertion into the complex. The covalent attachment of FMN (flavinylation) is catalyzed by the flavin transferase ApbE and was demonstrated for RNF from *V. cholerae* (Backiel et al. 2008), for RNF1 from *A. vinelandii* (Bertsova et al. 2021), and for the NQR from *V. harveyi* and *K. pneumoniae* (Bertsova et al. 2013). Moreover, the insertion of the intramembranous [2Fe-2S] into NQR seems to require the small membrane-bound iron-sulfur maturation protein NqrM in *V. cholerae* (Kostyrko et al. 2016; Agarwal et al. 2020). The genes coding for ApbE and NqrM are located on the *nqr* operon in *V. cholerae* downstream of the structural *nqrABCDEF* genes and are co-transcribed with the structural genes (Agarwal et al. 2020). The first structure of Na^+^-NQR in 2014 (Steuber et al. 2014) revealed that these aforementioned four cofactors, the intramembranous [2Fe-2S] cluster, the two covalently attached FMNs, and the riboflavin form an unexpected electron transfer pathway where the electron passes twice the cytoplasmic membrane. Electrons originating from an electron donor in the cytoplasm are transferred via these cofactors across the membrane to the periplasmic aspect and back to the electron acceptor at the cytoplasmic aspect (Fig. 1). This unusual pathway is, e.g., in stark contrast to complex I where the electron transfer chain is localized in the peripheral arm (reviewed in (Parey et al. 2020; Sazanov 2023)). Even more striking was the observation that several redox cofactors in the electron transfer chain reside too far from each other to allow for fast electron transfer. It became clear that several large conformational changes are required for the function of the complex (Steuber et al. 2014). Detailed information on these conformational changes became available only recently by new structural data on RNF and NQR (see also Table S1) in combination with kinetic analysis and site directed mutagenesis (Kishikawa et al. 2022; Vitt et al. 2022; Hau et al. 2023; Zhang and Einsle 2024; Kumar et al. 2025). In the following, we give a concise description of the structure of the individual subunits of RNF and NQR and describe their function in electron transfer and associated conformational changes starting from the electron donor subunit to the final electron acceptor subunit.

### The electron input module RnfB/NqrF

In the *forward electron transfer* mode, RnfB accepts electrons from ferredoxin (Fig. 1) and inserts these into the catalytic core formed by RnfAEDG. RnfB is composed of a single N-terminal TM helix and two hydrophilic, cytoplasmic domains. In the RnfB subunits structurally characterized so far, the central ferredoxin-like cytoplasmic domain is flexibly tethered to the TM helix and contains one [4Fe-4S] cluster. The C-terminal cytoplasmic domain differs among RNF from different organisms, comprising between two and seven [4Fe-4S] clusters (Table 1). This domain serves as acceptor for cytoplasmic ferredoxins and transfers the electrons via the small [4Fe-4S] ferredoxin domain to the membrane subunits RnfA-E. Interestingly, the small ferredoxin-like domain of RnfB positioned between the C-terminal domain of RnfB and subunits RnfA-E is not well resolved in the structures reported so far (Vitt et al. 2022; Zhang and Einsle 2024; Kumar et al. 2025), which probably reflects a high degree of flexibility of this domain.

During evolution of NQR the RnfB subunit was replaced by NqrF (Reyes-Prieto et al. 2014) that is, similar to RnfB, composed of a single TM helix, a small and flexible ferredoxin-like domain carrying a [2 Fe-2S] cluster, and a larger electron input domain. The latter comprises a FAD containing FNR-like domain whose FAD_NqrF_ accepts a hydride from NADH. NqrF is not related to the NADH-oxidizing subunit of the mitochondrial complex I (Parey et al. 2021) and its NADH-binding pocket is therefore well-suited for the development of new antibiotics (Kaminski et al. 2022). Electrons are shuttled from these electron donor modules RnfB and NqrF into the conserved energy-converting core machinery. The four membrane subunits of this core are organized as three functional units, which catalyze transmembrane electron transfer and couple it to Na^+^ or H^+^ translocation across the membrane as shown by mutational and functional studies (Juárez et al. 2010) and the first structure of the NQR complex (Steuber et al. 2014).

#### RnfA-RnfE/NqrE-NqrD

**Fig. 2:**
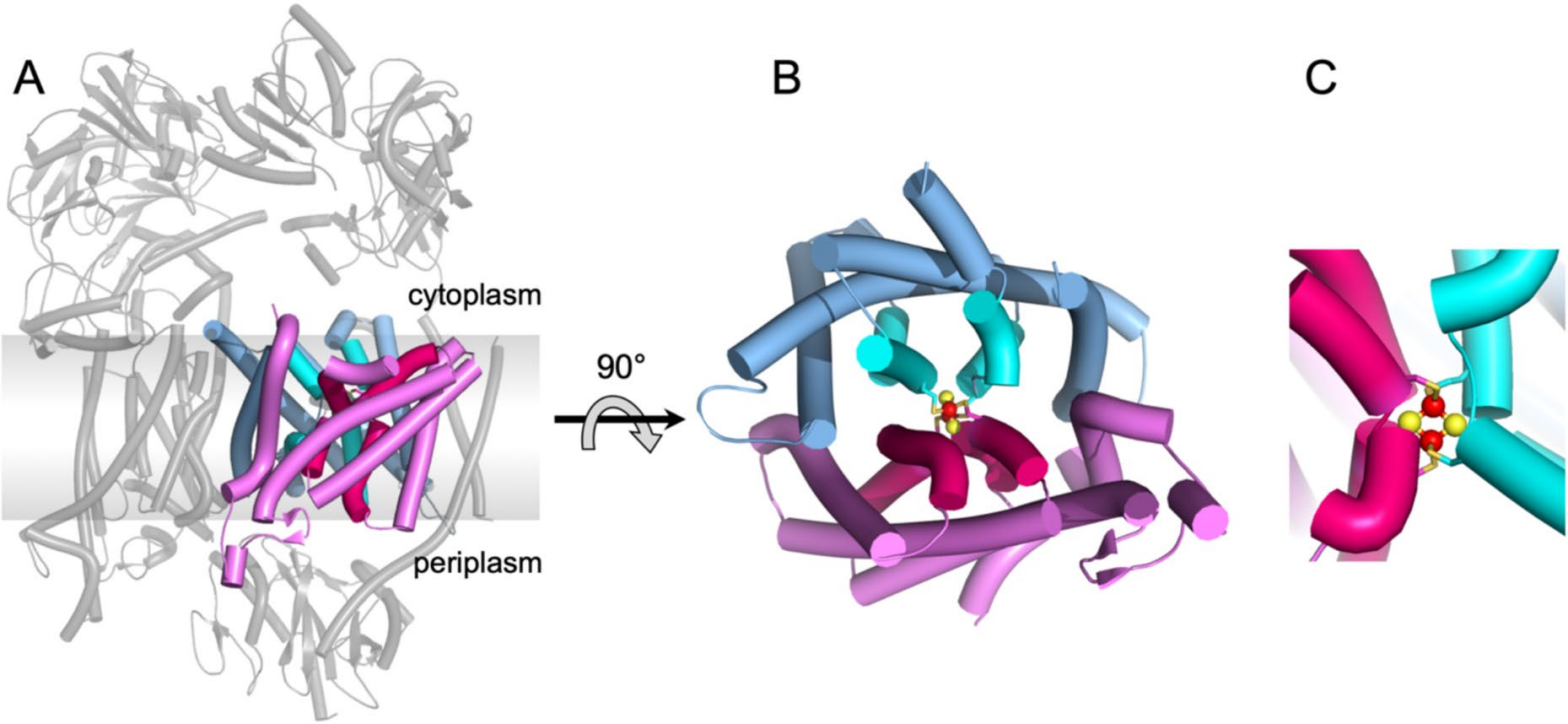
Architecture of the RnfA-E/NqrE-D dimer coordinating a membrane-bound [2Fe-2S] cluster. **A**, Structure of NQR of *V. cholerae* indicating the localization of NqrE (blue/cyan) and NqrD (magenta/purple) in NQR. The membrane plane is indicated by a grey box. **B**, View of the NqrE-D subunits from the periplasm. The inner helices of NqrE (cyan) and of NqrD (purple) each provide a Cys (in total 4) to coordinate the [2Fe-2S] cluster. C, Close-up view of the inner helices coordinating the [2Fe-2S] cluster.

**Fig. 3:**
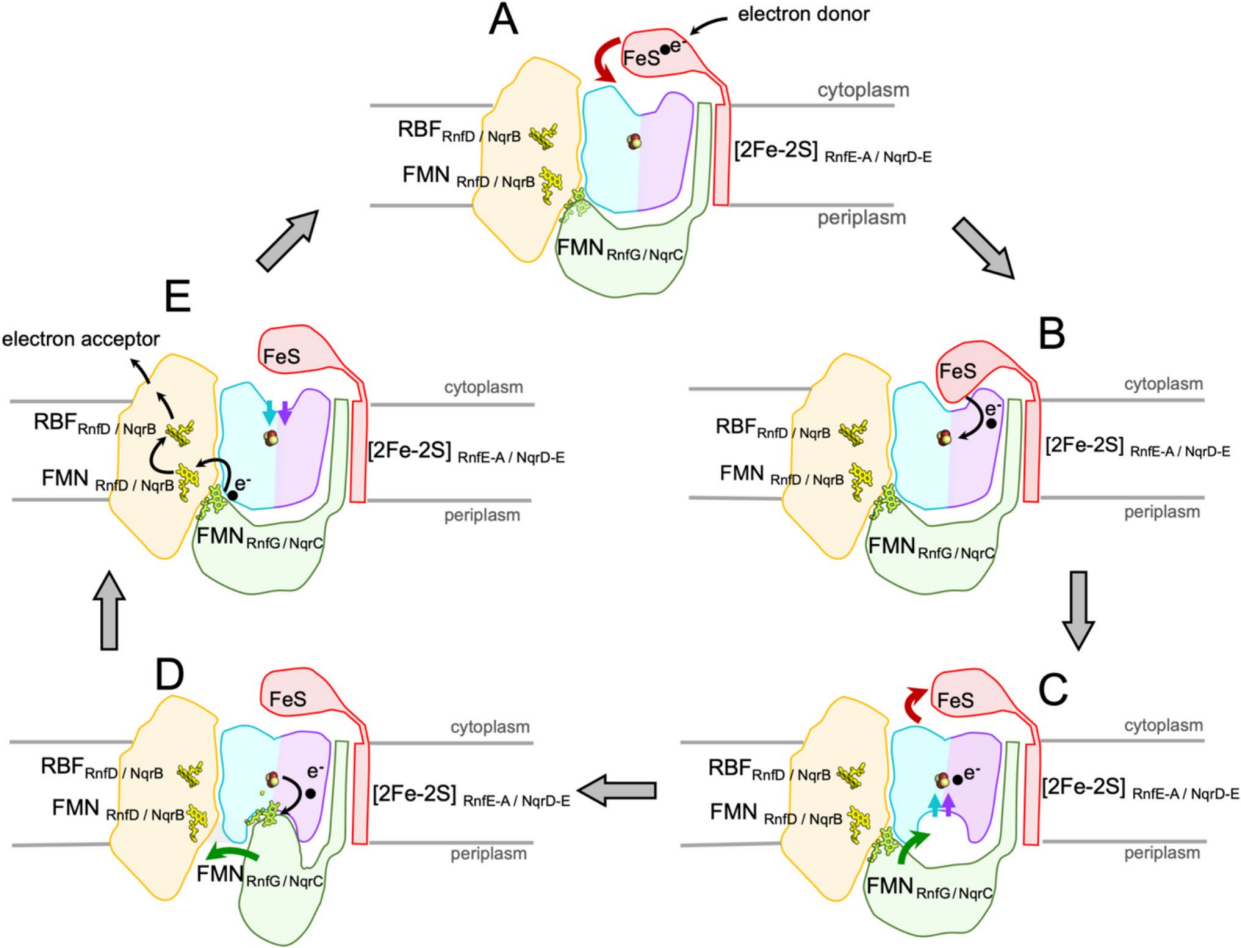
Conformational changes during electron transfer in RNF/NQR. **A** The unusual electron transfer in RNF/NQR through the membrane is initiated by the transfer of an electron to the ferredoxin-like domain of RnfB/NqrF (red). The RnfE-A/NqrD-E heterodimer (magenta/cyan) adopts an inward conformation that allows access of the intramembranous [2Fe-2S]_RnfE-A/NqrD-E_ cluster from the cytoplasmic side, whereas access for RnfG/NqrC (green) from the periplasmic/extracellular side is blocked. **B** The FeS cluster of the flexibly tethered ferredoxin-like domain of RnfB/NqrF can bind sufficiently close to [2Fe-2S]_RnfE-A/NqrD-E_ to rapidly transfer an electron. **C** The reduction of [2Fe-2S]_RnfE-A/NqrD-E_ triggers an inward-outward switch in subunits RnfE-A/NqrE-D, obstructing access to [2Fe-2S]_RnfE-A/NqrD-E_ from the cytoplasmic side, and facilitating access to [2Fe-2S]_RnfE-A/NqrD-E_ from the periplasmic/extracellular side. **D** RnfG/NqrC has shifted from RnfD/NqrB (yellow) to a position close to RnfE-A/NqrD-E and the electron is transferred from the [2Fe-2S]_RnfE-A/NqrD-E_ to FMN_RnfG/NqrC_. **E** The oxidation of [2Fe-2S]_RnfE-A/NqrD-E_ triggers the outward-inward switch and the rotation of RnfG/NqrC towards RnfD/NqrB. Subsequently, rapid electron transfer proceeds from FMN_RnfG/NqrC_ to FMN_RnfD/NqrB_, and from there to riboflavin (RBF) in RnfD/NqrB. From RBF_RnfD_ the electron is transferred to the proximal iron-sulfur cluster of subunit RnfC and from RBF_NqrB_, to ubiquinone

The first unit of the core machinery comprises a compact heterodimer of RnfA-RnfE/NqrE-NqrD that accepts a single electron from RnfB/NqrF and transfers the electron across the membrane to the periplasmic side as demonstrated by stopped-flow fast kinetics experiments for NQR (Hau et al. 2023). The structures of RnfA-E/NqrE-D are exceptionally well conserved (Table 1) reflected also by a high sequence identity between the RNF and NQR complexes (electronic supplementary material, Fig. S1). Interestingly, the *rnfA* and *rnfE* genes are result of a duplication of a single ancestor gene (Rapp et al. 2006). The structurally closely related encoded proteins are arranged in an inverted topology (Figs. 1, 2) (Sääf et al. 1999). Both subunits RnfA-RnfE or NqrE-NqrD consists each of six TM helices. As a whole, the RnfA-RnfE/NqrE-NqrD dimer exhibits eight outer TM helices that encircle four inner TM helices (Fig. 2). Notably, the inner four TM helices are interrupted by a short unstructured stretch splitting the helix into two half-helices. Each second half-helix contains a Cys residue, which together coordinate a [2Fe-2S] cluster (Fig. 2) in the center of the RnfA-RnfE/NqrE-NqrD dimer and in the middle of the membrane. This extraordinary cluster has been characterized in detail for NQR by various spectroscopic techniques (Hau et al. 2023) and its presence in RNF has been confirmed by recent structural data (Zhang and Einsle 2024; Kumar et al. 2025). The [2Fe-2S] cluster in RnfA-RnfE/NqrE-NqrD dimer accepts electrons from the cytoplasmic FeS cluster of the flexible ferredoxin domain of RnfB/NqrF and transfers these electrons across the membrane to the periplasmic side to the flavin containing RnfG/NqrC. This transmembrane electron transfer is controlled by conformational changes of the RnfA-E/NqrD-E dimer, which are likely controlled by the redox-state of the intramembranous [2Fe-2S] cluster (Hau et al. 2023). Two major conformations representing snapshots of the transmembrane electron transfer have been resolved for NqrD-E in several structures (Hau et al. 2023), which must occur also in RnfA-E. Based on the experimental data it is proposed that in the oxidized state the RnfA-E/NqrD-E dimer opens towards the cytoplasmic side and the flexible ferredoxin domain of RnfB or NqrF can access the [2Fe-2S] cluster. Upon reduction of the cluster, the conformation of the dimer changes and opens towards to the periplasmic side while it closes at the cytoplasmic side. At the periplasmic side, the subunit RnfG/NqrC can now reach the [2Fe-2S] cluster in RnfA-E/NqrD-E to accept the electron (Fig. 3) as documented by structural and cross linking data (Hau et al. 2023). Thus, there is alternating access of the cytoplasmic and periplasmic redox partners to the [2Fe-2S] cluster in RnfA-E/NqrD-E dependent on the redox state of the cluster.

#### RnfG/NqrC

**Fig. 4:**
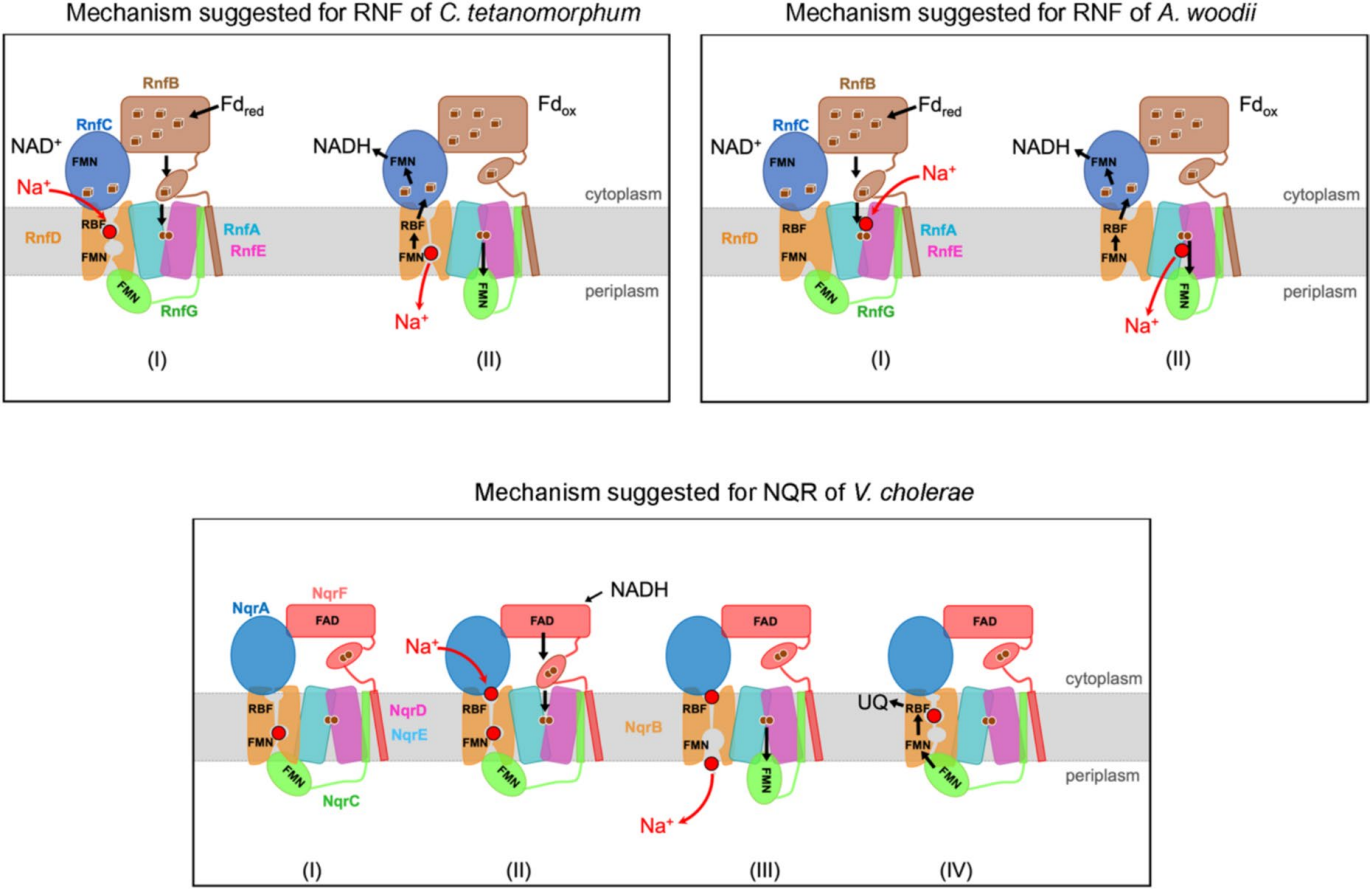
Proposed mechanisms of redox-driven Na^+^ transport by RNF and NQR. Conserved RNF/NQR subunits are presented with identical colors. In RNF operating in the *forward electron transfer* mode illustrated here, electrons are provided by reduced ferredoxin, while in NQR NADH serves as electron donor. Top, left: Proposed Na^+^ transport mechanism by RNF from *C. tetanomorphum* via a gated channel in RnfD. (I) The flexible reduced ferredoxin-like domain binds to the inward-facing state of RnfA-E, reduces its [2Fe-2S] cluster and fixes a Na^+^ in a strained state. (II) The RnfG shuttle moves to the outward-facing position of RnfA-E, thereby triggering pore opening at RnfD and Na^+^ passage. After reduction RnfG flips back to RnfD and induces a conformational change that leads to the release of Na^+^. The electron is transferred further to RnfC and NAD^+^. Top, right: Proposed Na^+^ transport mechanism by RNF from *A. woodii* via RnfA-E. (I) Na^+^ and the reduced RnfB ferredoxin-like domain bind to the inward-facing conformation. Upon [2Fe-2S]_RnfA-E_ reduction, an inward-to-outward switch translocates Na^+^ to the periplasm. The electron is shuttled via RnfG, RnfD and RnfC to NAD^+^. Bottom: Proposed mechanism for NQR from *V. cholerae* via NqrB (I) Na^+^ is bound in NqrB at the periplasmic half-channel, but release is blocked by NqrC. NqrD-E heterodimer opens to the cytoplasmic side. (II) An electron is transferred from NqrF to the membrane [2Fe-2S]_NqrD-E_ cluster. This triggers an inward-to-outward switch of NqrD-E and prompts Na^+^ binding to NqrB (III). NqrC moves from NqrB towards NqrD-E and Na^+^ is released from NqrB to the periplasm. (IV) NqrC switches back to NqrB. It is proposed that electron transfer to FMN_NqrB_ triggers translocation of the Na^+^ in NqrB. The electron is transferred via riboflavin_NqrB_ to ubiquinone. Starting again from state (I), the second electron derived from the FAD semiquinone in NqrF is injected into the core, and the cycle is repeated. Per NADH oxidized, one ubiquinol is formed and two Na^+^ are translocated

The second unit of the core machinery is represented by subunit RnfG/NqrC predominantly located at the extracellular/periplasmic membrane side. It is composed of a single N-terminal TM helix, a linker helix, and a large C-terminal globular α/β domain. The latter harbors as redox cofactor an FMN that is covalently attached to a conserved threonine (electronic supplementary material, Fig. S2) via a phosphodiester bond. The isoalloxazine ring of FMN is not fully embedded in the protein matrix and is partially exposed. The structures of RnfG and NqrC are reasonably well conserved, as documented by low RMSD values over the entire structure (Table 1); however, this is not reflected in sequence similarity. In structure-based alignments, the sequence identity is rather low, except for the regions in the proximity of the FMN. In particular conserved are residues 223–227 (NqrC *V. cholerae* numbering) containing the FMN-binding T225, residue K207 forming a salt bridge to the phosphate group of FMN, and residues 172–176 (Fig. S2), which form a binding pocket for the isoalloxazine ring. RnfG/NqrC shuttles the electron from RnfA-RnfE/NqrE-NqrD to RnfD/NqrB as proposed first for NQR (Steuber et al. 2014) bridging an unusually large distance of approximately 35 Å between the redox cofactors of these subunits (Figs. 3, 4). Electron transfer across such large distances is not feasible and is overcome by a large conformational change of RnfG/NqrC switching between RnfA-RnfE/NqrE-NqrD and RnfD/NqrB. For NQR, it was shown that the hydrophilic domain of NqrC undergoes a rotational movement by approximately 25–30° (Fig. 4). A combined approach using mutagenesis, cross-linking by engineered cysteines, and mass spectrometric studies together with X-ray and cryo-EM structures demonstrated that NqrC moves during catalysis between NqrD/NqrE and NqrB (Hau et al. 2023). Similar switching movements have been proposed for the homologous RnfG (Zhang and Einsle 2024; Kumar et al. 2025). Thus, RnfG/NqrC acts as an electron transfer switch module controlling electron transfer between [2Fe-2S]_RnfAE/NqrED_ and FMN_RnfD/NqrB_. Notably, the cryo-EM structures determined so far of RNF or NQR complexes revealed RnfG/NqrC primarily located at RnfD/NqrB, while in the crystal structure of NQR the soluble domain of NqrC was positioned at the NqrDE dimer (Steuber et al. 2014) most likely stabilized by crystal contacts. These findings put forward that the localization of subunit RnfG/NqrC at RnfD/NqrB is energetically favored or stabilized and that the movement towards RnfA-RnfE/NqrE-NqrD heterodimer requires energy. However, the energetics causing these large movements of RnfG/NqrC are not yet understood and are subject of further investigations.

#### RnfD/NqrB

The third unit of the core machinery is subunit RnfD/NqrB, which represents with 10 TM helices the largest integral membrane subunit of RNF and NQR (Figs. 1, 3). Remarkably, RnfD/NqrB is structurally and evolutionary related to the ammonium/urea transporter family that also translocate cations or solutes across the membrane (Andrade et al. 2005; Levin et al. 2012). In contrast to the solute transporters, RnfD/NqrB contains FMN and riboflavin as redox cofactors. Riboflavin as a redox cofactor is rather unique to RNF and NQR and has not been observed so far in other flavin enzymes or respiratory complexes. While the riboflavin is localized closer to the cytoplasmic side, FMN is located in proximity to the periplasmic side and is covalently bound via a phosphodiester bond to a threonine (electronic supplementary material, Fig. S3). The electron is transferred from the FMN of the switch module RnfG/NqrC to FMN_RnfD/NqrB_, from which it rapidly flows to riboflavin_RnfD/NqrB_ about 8 Å apart. The further electron transfer from riboflavin_RnfD/NqrB_ to the terminal electron acceptor differs in RNF and NQR. In RnfD, the electron is shuttled from riboflavin_RnfD_ to RnfC, the site of the terminal acceptor NAD^+^. In NqrB, the electron from riboflavin_NqrB_ directly flows to the terminal electron acceptor ubiquinone bound to NqrB (Hau et al. 2023). The quinone binding site is not located within the protein core like, e.g., in complex I, but in a pocket at the protein surface. The binding site is formed by two further N-terminal amphipathic helices of NqrB (Hau et al. 2023) not present in RnfD (Fig. S3). Several inhibitors like 2-heptyl-4-hydroxyquinoline-N-oxide (HQNO) (Hau et al. 2023), aurachin D-42, and korormicin A (Kishikawa et al. 2022) bind to this quinone-binding site of NqrB.

### The electron output module RnfC

RnfC catalyzes NAD^+^ reduction in RNF operating in *forward electron transfer mode* and contains one FMN and 2 [4Fe-4S] cofactors (Fig. 1). The electron output module RnfC is primarily composed of a larger FMN-carrying domain that is related to the FMN containing NuoF of complex I, and a small C-terminal ferredoxin domain carrying two [4Fe-4S] clusters. The two [4Fe-4S] clusters bridge the riboflavin of RnfD inside the membrane (Fig. 1) to the FMN of RnfC (Vitt et al. 2022; Zhang and Einsle 2024; Kumar et al. 2025) and after the transfer of two electrons a hydride is donated from reduced FMN to NAD^+^ (Kuhns et al. 2020a). In contrast to RnfC of *C. tetanomorphum* or of *A. woodii*, RnfC of *A. vinelandii* displays a C-terminal elongated helix of more than 50 residues length, which interacts with RnfB and RnfH. The exact function of this long helix is not yet understood.

The subunit NqrA of NQR is highly homologous and structurally similar to RnfC; however, it does not contain any redox cofactors and cannot be assigned a direct function. We have speculated that NqrA might be required to stabilize the membrane subunit NqrB or the position of NqrF in the cytoplasm (Hau et al. 2023), but other functions cannot be ruled out.

### Mechanism of redox-driven ion transport in RNF/NQR complexes

In light of the evolutionary relationship and high structural similarity between RNF and NQR, it is likely that both complexes operate via a similar mechanism for redox-driven ion translocation. The unique architecture of RNF/NQR compared to other well characterized respiratory complexes implies that ion translocation must be quite different from other so far described mechanisms, e.g., for complex I (Parey et al. 2020; Sazanov 2023). The unusual transmembrane electron transfer pathway and the large conformational changes linked to electron transfer in RNF/NQR clearly set them apart from other known respiratory complexes. This has been proposed already by the results from former biochemical, spectroscopic, and mutational studies on the redox reactions, the ion transport reaction and the coupling of both reactions as summarized in previous reviews (Verkhovsky and Bogachev 2010; Biegel et al. 2011; Juárez and Barquera 2012; Buckel and Thauer 2013; Steuber et al. 2015) (see also Table S2). And finally, considering a time span of at least 1 billion years of separate evolution of RNF and NQR (Reyes-Prieto et al. 2014; Munoz et al. 2020; Ward and Shih 2022) one cannot rule out some differences in the mechanism of these complexes.

While we meanwhile have very good evidence for the electron transfer steps and can provide a reasonable model, the ion transport process and the coupling between both processes is not fully understood yet and a matter of debate. So far, different putative mechanisms have been suggested for RNF and NQR (Fig. 4), nevertheless all mechanistic scenarios of RNF and NQR are based on common principles: Exergonic electron transfer steps and the endergonic ion translocation are indirectly coupled via various structural rearrangements.

In the mechanism suggested for NQR and for RNF from *C. tetanomorphum*, a central role of RnfD/NqrB is proposed (Vitt et al. 2022; Hau et al. 2023). Comparative structural studies revealed a close structural and evolutionary relationship between RnfD/NqrB and the ammonium/urea solute transporter family marking RnfD/NqrB as a plausible candidate for ion translocation. Of note, in the structures available so far, the observed conformational changes in RnfD/NqrB are rather small compared to the changes in other subunits. Nevertheless, comparison of RnfD/NqrB with the solute transporters revealed several differences. The structure of the solute transporters exhibits a continuous channel for the transported molecule. In contrast, in RnfD/NqrB, a putative ion pathway is characterized by a constriction blocking a continuous flow, which is required for a pumping mechanism. This constriction site is mainly formed by the bulky hydrophobic side chains of F338 and F342 (NqrB numbering), which might act as a gate during ion translocation. Mutation of either F338 or F342 to a smaller alanine resulted in 30% lower voltage formation for NQR. This might be caused by a backflow of Na^+^ due to incomplete closure of the proposed gate (Hau et al. 2023). The pathway through NqrB also includes a *bona fide* binding site of the coupling ion Na^+^ (Hau et al. 2023) localized between the constriction and a proposed periplasmic exit site. Since Na^+^ and water exhibit very similar density, Na^+^ can only be identified by the higher coordination number and the coordination geometry. In NqrB, the Na^+^ is coordinated by backbone carbonyl oxygens of A263, V275, V332 (Fig. S3) and two water molecules (Hau et al. 2023). Interestingly, in *A. vinelandii* RNF, the protonatable sidechain of glutamate E219 resides at the position of the Na^+^ in NQR, suggesting this site could allow binding of a proton instead of a Na^+^. *C. tetanomorphum* RnfD contains an alanine at this position, compatible with Na^+^ coordination (Fig. S3). As described in Fig. 4, the energy to convert the relaxed closed into the strained open state is presumably provided by Na^+^ binding (Vitt et al. 2022) and, in particular, by the conformational changes of the ferredoxin domain of RnfB/NqrF and RnfG/NqrC propagated to the constriction.

In a further mechanism recently described for RNF of *A. woodii* (Kumar et al. 2025), Na^+^ translocation is proposed to proceed across the RnfA-E heterodimer. Although the RnfA-E/NqrE-D have no structural homologue, its overall architecture with half helices in the core is reminiscent of H^+^/solute and Na^+^/solute symporters operating via an alternating access mechanism between inward-facing and outward-facing conformations (Gotfryd et al. 2020). Like H^+^/solute and Na^+^/solute symporters, the RnfA-E/NqrE-D dimer exhibits two different conformations, where the structure opens either to the cytoplasmic aspect or to the periplasmic aspect. Interestingly, the closure in the RnfA-E/NqrE-D dimer is in the midst of the membrane next to the [2Fe-2S] cluster. Kumar et al. (2025) proposed a conformational change in the RnfA-E dimer during electron transfer that allows a Na^+^ to shuttle across the membrane and supported this hypothesis by mutations and elaborate molecular dynamics studies.

The large conformational changes in RNF/NQR and the suggested mechanisms set these complexes apart from so far described respiratory enzymes and illustrate the different solutions nature has elaborated to couple redox reactions and transport processes. The recent studies have revealed many fascinating and unique properties of RNF/NQR, but future studies are needed to answer open questions and shed more light into the details of these remarkable molecular machines. New approaches might provide a comprehensive picture of putative further conformations of RNF/NQR and of the redox states coupled to distinct steps of ion translocation.

## Structure and function of RNF and NQR: an outlook

Ferredoxins are primordial redox carriers, which link the central metabolism to ATP regeneration in many anaerobes. RNF plays a crucial role in pathways of energy conservation, which have occurred early in evolution, as it couples the oxidation of reduced ferredoxin to the build-up of an electrochemical Na^+^ (or H^+^) gradient driving ATP synthesis. RNF also provides reduced ferredoxin for energy-demanding processes such as N_2_ fixation, defining it as a versatile bioenergetic machine for reduction or oxidation of ferredoxins. Understanding the structure, function, and mechanism of RNF opens opportunities to optimize biotechnological processes and modulate the redox state of the ferredoxin pool in a cell with the help of RNF. Applying metabolic engineering could, e.g., potentially drive conversion of CO_2_ into biofuels in acetogenic bacteria (Katsyv and Müller 2020). Moreover, RNF and NQR are essential respiratory enzymes in many pathogens like pathogenic clostridia or highly virulent and multi-drug resistant *Klebsiella pneumoniae*,* Acinetobacter baumannii*, and *Pseudomonas aeruginosa*, which are top listed in the WHO Bacterial Priority Pathogens List 2024 (Sati et al. 2025). The now available structures of these complexes represent an excellent basis for structure-based drug design to obtain novel, specific inhibitors against these pathogens, for which new antibiotics are urgently needed.

## Supplementary Information

Below is the link to the electronic supplementary material.

Supplementary Material 1 (DOCX 1.23 MB)

## Data Availability

No datasets were generated or analysed during the current study.

## Abbreviations

RNF: Rhodobacter nitrogen fixation complex (Na^+^ or H^+^ -translocating ferredoxin:NAD^+^ oxidoreductase)
RSX: Reducing system for SoxR
NQR: Na^+^ or H^+^ -translocating NADH:quinone oxidoreductase
Rbf: Riboflavin
UQ: Ubiquinone
UQH_2_: Ubiquinol
