# Supplementary material for: The RNF/NQR redox pumps: a versatile system for energy transduction in bacteria and archaea
Source: Appl Microbiol Biotechnol. 2025 Jun 17;109(1):148. doi: 10.1007/s00253-025-13531-0 (PMC12174285; doi:10.1007/s00253-025-13531-0)
Supplement: Supplementary file 1 — Supplementary Material 1 (DOCX 1.23 MB) [file 253_2025_13531_MOESM1_ESM.docx]

**Electronic supplementary material**

The RNF/NQR redox pumps: A versatile system for energy transduction in Bacteria and Archaea

Wolfgang Buckel^1^, Ulrich Ermler^2^, Janet Vonck^3^, Günter Fritz^4^, Julia Steuber^4^*

^1^Philipps-Universität Marburg, Faculty of Biology, Karl-von-Frisch-Straße 8, 35043 Marburg, Germany.

^2^Department of Molecular Membrane Biology, Max Planck Institute of Biophysics, Max-von-Laue-Straße 3, 60438 Frankfurt am Main, Germany.

^3^Department of Structural Biology, Max Planck Institute of Biophysics, Max-von-

Laue-Straße 3, 60438 Frankfurt am Main, Germany

^4^Department of Cellular Microbiology, Institute of Biology, University of Hohenheim, Garbenstraße 30, 70599 Stuttgart, Germany

*corresponding author

[julia.steuber@uni-hohenheim.de](mailto:julia.steuber@uni-hohenheim.de)

phone +49 711 459 22228

fax +49 711 459 22238


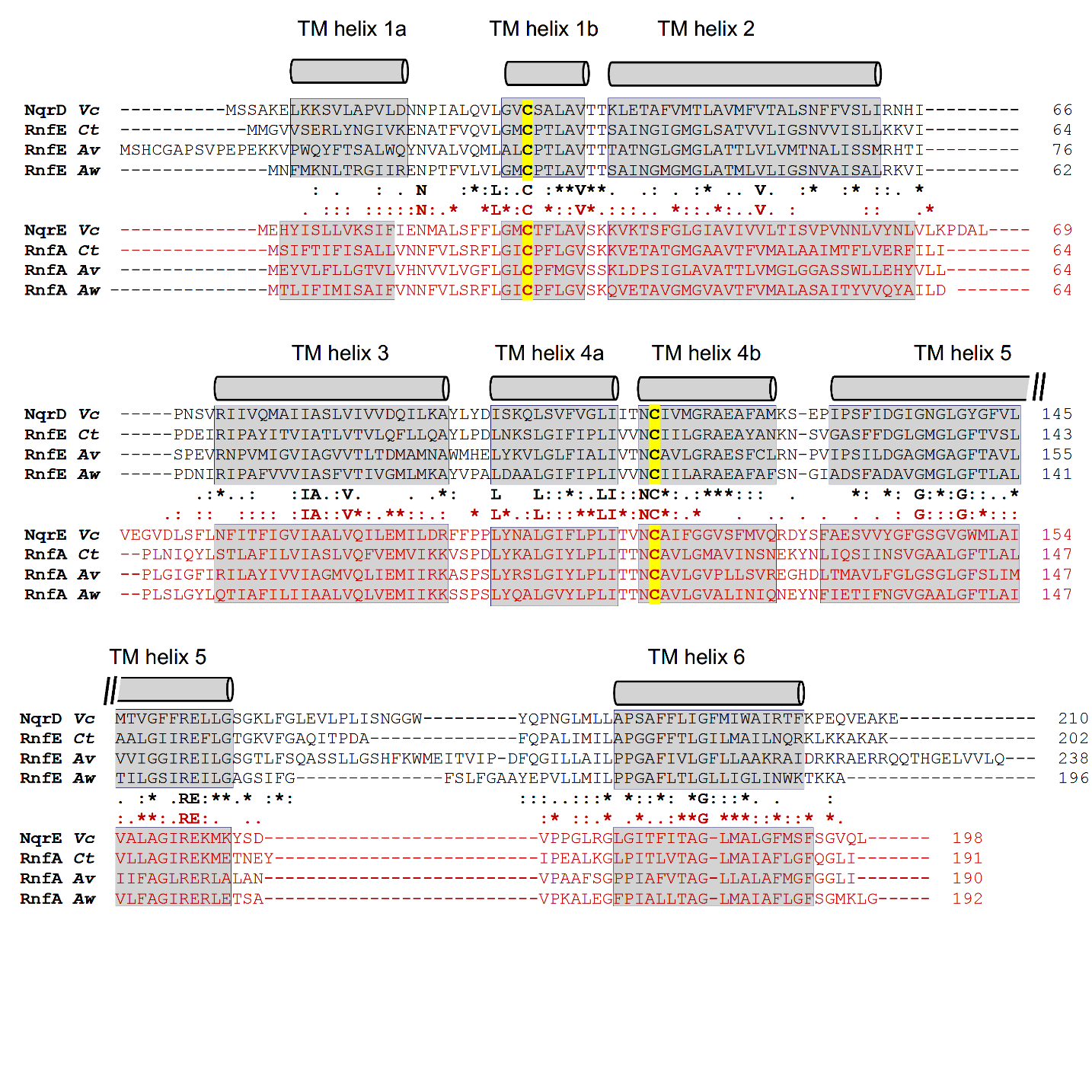


**Figure S1: Structure-based sequence alignment of the homologous subunits NqrD/RnfE and NqrE/RnfA.**

Black, NqrD/RnfE; red, NqrE/RnfA. Coordinates for alignment were NQR from *Vibrio cholerae* (pdb code 8a1u)*, Clostridium tetanomorphum* (pdb code 7zc6), RNF1 from *Azotobacter vinelandii* (pdb code 8ahx) and RNF from *Acetobacterium woodii* (9erk). Transmembrane helices are highlighted in grey. Cysteine residues coordinating the [2Fe-2S] cluster are highlighted in yellow. Consensus for NqrD/RnfE and NqrE/RnfA is indicated in black and red, respectively. Fully conserved residues in both proteins are shown in bold font. Structural alignments were prepared using ChimeraX (Meng et al. 2023).


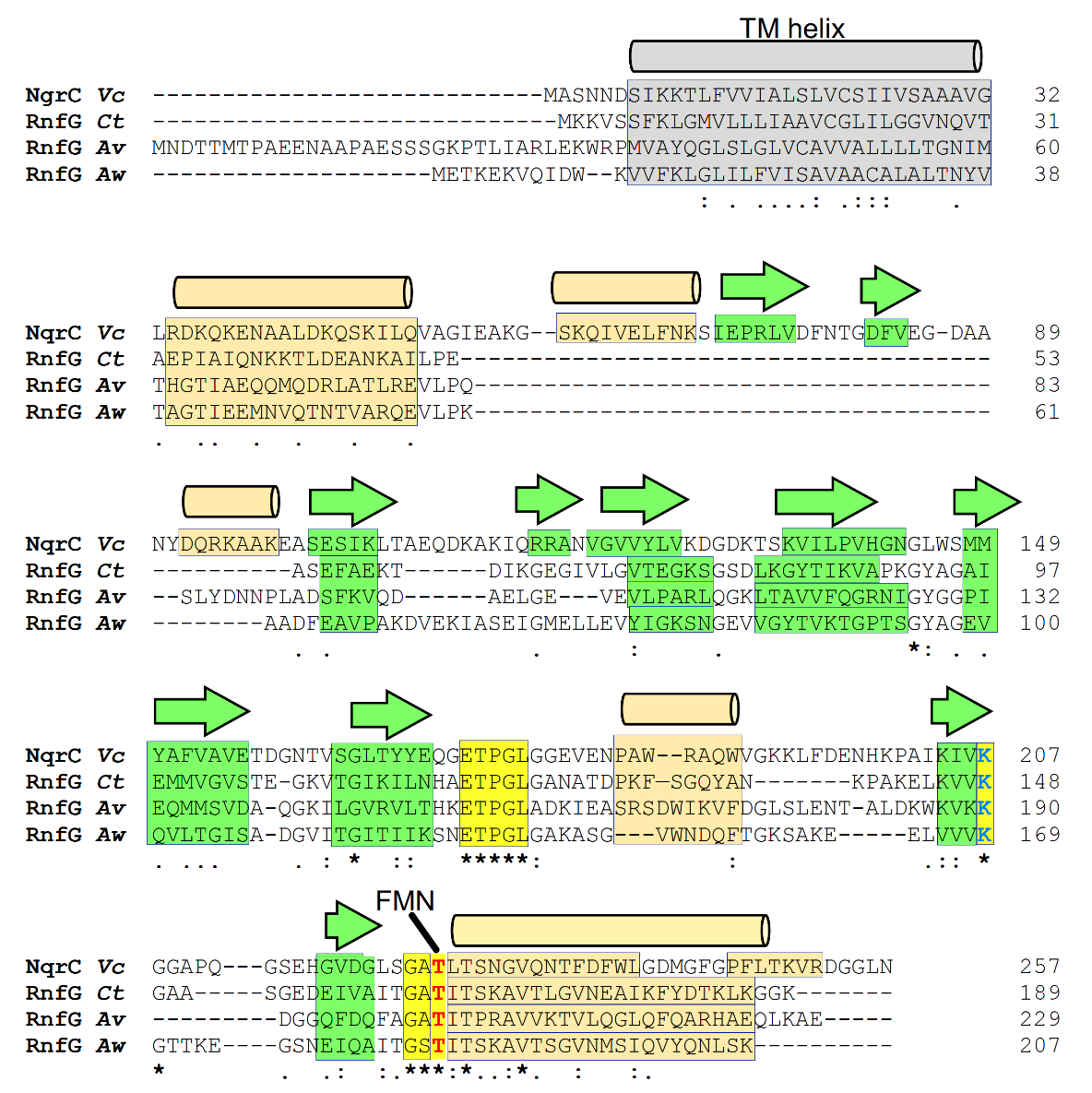


**Figure S2: Structure-based sequence alignment of the homologous subunits NqrC and RnfG.**

Coordinates for alignment were NQR from *Vibrio cholerae* (pdb code 8a1u), from *Clostridium tetanomorphum* (pdb code 7zc6), from *Azotobacter vinelandii* (pdb code 8ahx) and RNF from *Acetobacterium woodii* (9erk). Grey: the single transmembrane helix; beige: other α helix;  green: β strand. The conserved Thr for covalent attachment of FMN is highlighted in red. Conserved residues in the FMN binding pocket are highlighted in yellow. The conserved K207 (yellow, blue letter) (*Vibrio cholerae* NqrC numbering) coordinates the phosphate of FMN. Structural alignments were prepared using ChimeraX (Meng et al. 2023).


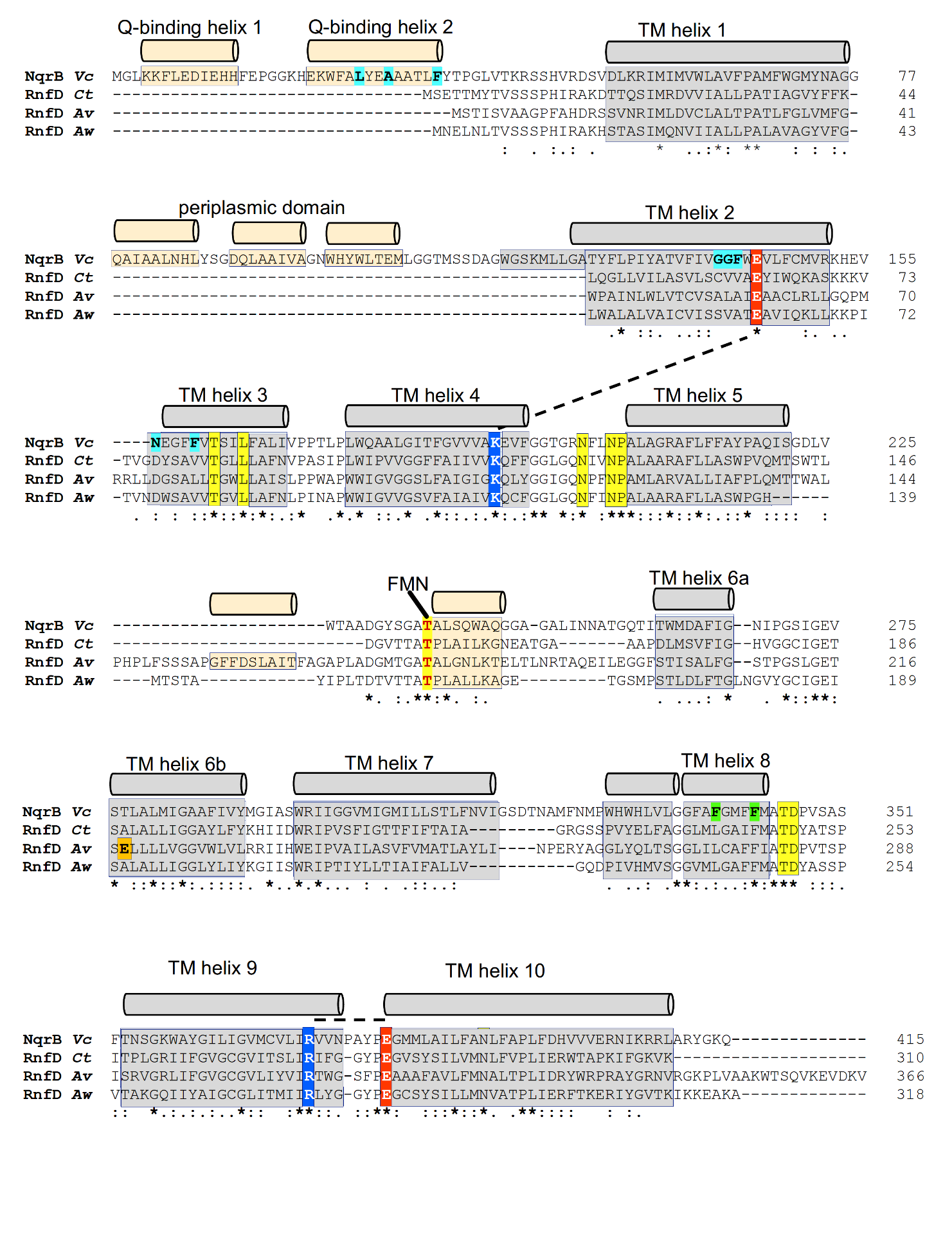


**Figure S3: Structure-based sequence alignment of the homologous subunits NqrB and RnfD**

Coordinates for alignment were NQR from *Vibrio cholerae* (pdb code 8a1u), from *Clostridium tetanomorphum* (pdb code 7zc6), from *Azotobacter vinelandii* (pdb code 8ahx) and RNF from *Acetobacterium woodii* (9erk). Grey: transmembrane helices (numbered); beige: other helices. Residues important for function are highlighted: Thr covalently linked to FMN (yellow, red font); residues participating in non-covalent binding of riboflavin (yellow, black font); residues participating in binding of quinone in *V. cholerae* NqrB (cyan). The aromatic sidechains of F338 and F342 (green) in TM helix 8 form a constriction of a putative Na^+^ translocation pathway in NqrB. In proton-gradient driven *A. vinelandii*, the sidechain of E219 (orange) resides at the position of a Na^+^ ion in NQR. In Na^+^-pumping complexes an Ala or Thr resides at this position. Two salt bridges which are conserved in RNF and NQR (E144 - K191 and R372 - E380) are highlighted (red and blue, white font). *V. cholerae* NQR numbering is used unless otherwise indicated.

**Table S1: Compilation of structural information on RNF and NQR complexes.**

Complexes, subunits or protein domains from *Acetobacterium woodii*, *Clostridium tetanomorphum,* *Azotobacter vinelandii*, *Vibrio cholerae*, *Vibrio harveyi*, *Pseudomonas aeruginosa*, *Klebsiella pneumoniae*, *Shewanella oneidensis*.

| Complex  /protein | PDB  /EMDB code | Organism | Subunit(s) or domain | Compound added;  Substrates or cations identified | Resolution [Å]  (method) | Reference |
| --- | --- | --- | --- | --- | --- | --- |
| **RNF** |  |  |  |  |  |  |
| RNF | 9ERL/  19920 | *A.woodii* | RnfABCDEG | As isolated | 3.00(cryo-EM) | (Kumar et al. 2025) |
| RNF | 9ERI/  19915 | *A.woodii* | RnfABCDEG | Treated with NADH | 3.30 (cryo-EM) | (Kumar et al. 2025) |
| RNF | 9ERK/  19919 | *A.woodii* | RnfABCDEG | With reduced ferredoxin state 1 | 2.80 (cryo-EM) | (Kumar et al. 2025) |
| RNF | 9ERJ/  19916 | *A.woodii* | RnfABCDEG | With reduced ferredoxin state 2 | 2.90 (cryo-EM) | (Kumar et al. 2025) |
| RNF | 8AHX/ 15452 | *A.vinelandii* | RnfABCDEGH | (native) | 3.11 (cryo-EM) | (Zhang and Einsle 2024) |
| RNF | 8RB9/ 19029 | *A.vinelandii* | RnfABCDEGH | NADH | 3.41 (cryo-EM) | (Zhang and Einsle 2024) |
| RNF | 8RB8/ 19028 | *A.vinelandii* | RnfABCDEGH | NADH + β-mercapto-ethanol + TCEP (tris(2-carboxyethyl)-phosphine) | 3.19 (cryo-EM) | (Zhang and Einsle 2024) |
| RNF | 8RBQ / 19034 | *A.vinelandii* | RnfABCDEGH | NADH + Na_2_S_2_O_4_ | 3.24 (cryo-EM) | (Zhang and Einsle 2024) |
| RNF | 8RBM /19032 | *A.vinelandii* | RnfABCDEGH | K_3_Fe(CN)_6_ | 3.32 (cryo-EM) | (Zhang and Einsle 2024) |
| RNF | 7ZC6  /14622 | *C. tetano-morphum* | RnfABCDEG | (native) | 4.27 (cryo-EM) | (Vitt et al. 2022) |
| **NQR** |  |  |  |  |  |  |
| NQR | 8ACY | *V. cholerae* | NqrABCDEF | (native);  1 Na^+^ | 3.5 (X-ray) | (Hau et al. 2023) |
| NQR | 8ACW | *V. cholerae* | NqrABCDEF | (native);  1 K^+^ | 3.4 (X-ray) | (Hau et al. 2023) |
| NQR | 8A1T  /15088 | *V. cholerae* | NqrABCDEF | (native);  1 Na^+^  1 K^+^ | 3.37 (cryo-EM) | (Hau et al. 2023) |
| NQR | 8A1W  /15091 | *V. cholerae* | NqrABCDEF | ubiquinone-1;  2 Na^+^ | 2.56 (cryo-EM) | (Hau et al. 2023) |
| NQR | 8A1V  /15090 | *V. cholerae* | NqrABCDEF | ubiquinone-2;  2 Na^+^ | 2.73 (cryo-EM) | (Hau et al. 2023) |
| NQR | 8A1U  /15089 | *V. cholerae* | NqrABCDEF | NADH + ubiquinone-2;  2 Na^+^ | 2.86 (cryo-EM) | (Hau et al. 2023) |
| NQR | 8A1X  /15092 | *V. cholerae* | NqrABCDEF | 2-decyl-4-quinazolinyl amine (DQA) | 3.2 (cryo-EM) | (Hau et al. 2023) |
| NQR | 8ADO | *V. cholerae* | NqrABCDEF | DQA;  1 Na^+^  1 K^+^  1 Br^-^ | 3.1 (X-ray) | (Hau et al. 2023) |
| NQR | 8A1Y  /15093 | *V. cholerae* | NqrABCDEF | 2-heptyl-4-hydroxyquinoline-N-oxide (HQNO) | 3.3 (cryo-EM) | (Hau et al. 2023) |
| NqrF_129-408_ | 8AD4 | *V. cholerae* | C-terminal domain of NqrF | NADH | 1.5 (X-ray) | (Hau et al. 2023) |
| NqrF_129-408_ (F406A) | 8AD3 | *V. cholerae* | C-terminal domain of NqrF | (native) | 1.55 (X-ray) | (Hau et al. 2023) |
| NqrF_129-408_ (F406A) | 8AD5 | *V. cholerae* | C-terminal domain of NqrF | NADH | 1.65 (X-ray) | (Hau et al. 2023) |
| NQR | 7XK3  /33242 | *V. cholerae* | NqrABCDEF | (native) | 3.1 (cryo-EM) | (Kishikawa et al. 2022) |
| NQR | 7XK4  /33243 | *V. cholerae* | NqrABCDEF | (native) | 3.1 (cryo-EM) | (Kishikawa et al. 2022) |
| NQR | 7XK5  /33244 | *V. cholerae* | NqrABCDEF | (native) | 3.1 (cryo-EM) | (Kishikawa et al. 2022) |
| NQR | 7XK6  /33245 | *V. cholerae* | NqrABCDEF | Aurachin D-42 | 3.0 (cryo-EM) | (Kishikawa et al. 2022) |
| NQR | 7XK7  /33246 | *V. cholerae* | NqrABCDEF | Korormicin A | 2.9 (cryo-EM) | (Kishikawa et al. 2022) |
| NqrF_130-407_ | 7QU5 | *P. aeruginosa* | C-terminal domain of NqrF | 5-methyloxolan-2-on | 1.25 (X-ray) | (Kaminski et al. 2022) |
| NqrF_130-407_ | 7QU3 | *P. aeruginosa* | C-terminal domain of NqrF | 4-[(1H-1,3-benzodiazol-1-yl)methyl] benzonitrile | 1.6 (X-ray) | (Kaminski et al. 2022) |
| NqrF_129-407_ | 7QTY | *K. pneumoniae* | C-terminal domain of NqrF | 3-[(furan-2-yl)methyl]-1-(2-methylphenyl) thiourea | 1.69 (X-ray) | (Kaminski et al. 2022) |
| NqrF_129-407_ | 7QU0 | *K. pneumoniae* | C-terminal domain of NqrF | N-(2,6-difluorophenyl) acetamide | 1.62 (X-ray) | (Kaminski et al. 2022) |
| NqrC_32-265_ | 4XHF | *S. oneidensis* | NqrC lacking N-terminal helix | (native) | 1.78 (X-ray) | (Deka et al. 2016) |
| NqrC_33–261_ | 4XA7 | *V. harveyi* | NqrC lacking N-terminal helix | (native) | 1.56 (X-ray) | (Borshchevskiy et al. 2015) |
| NQR | 4P6V (obsolete entry; refined structure deposited in 8ACY) | *V. cholerae* | NqrABCDEF | (native) | 3.5 (X-ray) | (Steuber et al. 2014) |
| NqrA_1-377_ | 4U9O | *V. cholerae* | NqrA lacking C-terminal helical domain | (native) | 1.60 (X-ray) | (Steuber et al. 2014) |
| NqrA_1-377_ | 4U9Q | *V. cholerae* | NqrA lacking C-terminal helical domain | (native) | 1.60 (X-ray) | (Steuber et al. 2014) |
| NqrC_33-257_ | 4U9S | *V. cholerae* | NqrC lacking N-terminal helix | (native) | 1.70 (X-ray) | (Steuber et al. 2014) |
| NqrF_129-408_ | 4U9U | *V. cholerae* | C-terminal domain of NqrF | (native) | 1.55 (X-ray) | (Steuber et al. 2014) |
| NqrF_129-408_ | 4UAJ | *V. cholerae* | C-terminal domain of NqrF | (native) | 2.7 (X-ray) | (Steuber et al. 2014) |

**Table S2: Biochemical studies of RNF and NQR complexes.**

Functional characterizations of RNF and NQR were performed using different methods, as exemplified by selected studies. Important experimental observations are highlighted. For comprehensive descriptions of functional properties of RNF and NQR, the reader is referred to the original publications. RNF from *Acetobacterium woodii*, *Thermotoga maritima* and *Clostridium tetanomorphum,* and NQR from *Vibrio cholerae* and *Vibrio harveyi* were studied.

| Complex  /protein | Organism | Sample analyzed | Experimental approach | Functional property | Reference |
| --- | --- | --- | --- | --- | --- |
| **RNF** | | | | | |
| RNF | *A. woodii* | Purified RNF | Steady-state kinetic analysis followed by VIS spectroscopy | Ferredoxin_red_: NAD^+^ oxido-reduction | (Kumar et al. 2025) |
| RNF | *C. tetano-morphum* | Purified RNF | Steady-state kinetic analysis followed by VIS spectroscopy | Ferredoxin_red_: NAD^+^ oxido-reduction | (Vitt et al. 2022) |
| RNF | *T. maritima* | Purified RNF (co-reconstituted in liposomes with F1FO ATP synthase) | Steady-state kinetic analysis followed by VIS spectroscopy  Na^+^ translocation (followed as Na^+^ gradient formation) | Ferredoxin_red_: NAD^+^ oxido-reduction  Electrogenic and primary Na^+^ transport | (Kuhns et al. 2020) |
| RNF | *A. woodii* | Membrane vesicles | Steady-state kinetic analysis followed by VIS spectroscopy  Na^+^ translocation (followed as Na^+^ gradient formation) | Ferredoxin_red_: NAD^+^ oxido-reduction  Electrogenic and primary Na^+^ transport | (Westphal et al. 2018)  (Biegel and Müller 2010)  (Imkamp et al. 2007) |
| **NQR** | | | | | |
| NQR | *V. cholerae* | Purified NQR | Steady-state kinetic analysis followed by VIS spectroscopy  Stopped-flow kinetic analysis followed by VIS spectroscopy  Chemical cross linking with mass spectrometry | NADH:Q oxido-reduction  Intra-molecular electron transfer [2Fe-2S]_NqrF_ → [2Fe-2S]_NqrDE_  Conformational change of NqrC | (Hau et al. 2023) |
| NQR | *V. cholerae* | Purified NQR (reconstituted in liposomes) | Na^+^ translocation (followed as transmembrane voltage formation) | Na^+^ transport | (Hau et al. 2023) |
| NQR | *V. cholerae* | Purified NQR | Kinetic analysis followed by VIS spectroscopy | K_M_ values for ubiquinone-1, NADH, Na^+^ | (Tuz et al. 2015) |
| NQR | *V. cholerae* | Purified NQR (reconstituted in liposomes) | Na^+^ translocation (followed as Na^+^ gradient formation) | Na^+^ transport | (Muras et al. 2014) |
| NQR | *V. cholerae* | Purified NQR  (reconstituted in liposomes) | Na^+^ translocation, Li^+^ translocation (followed as transmembrane voltage formation) | Na^+^ transport,  Li^+^ transport | (Juárez et al. 2011) |
| NQR | *V. cholerae* | Purified NQR | Kinetic analysis  monitored by VIS and EPR spectroscopy | Inter-molecular electron transfer riboflavin_NqrB_ → Q | (Juárez et al. 2008) |
| NQR | *V. harveyi* | Purified NQR | Stopped-flow kinetic analysis  monitored by VIS and EPR spectroscopy | Very fast initial electron transfer (Na^+^ -independent): NADH → FAD _NqrF_  → [2Fe-2S]_NqrF_  Intermediate phase (Na^+^ stimulated):  Formation of neutral flavosemiquinone  Slow phase (Na^+^ stimulated):  Formation of anionic flavosemiquinone | (Bogachev et al. 2009)  (Bogachev et al. 2002)  (Bogachev et al. 2001) |

References:

Biegel E, Müller V (2010) Bacterial Na ^+^ -translocating ferredoxin:NAD ^+^ oxidoreductase. Proc Natl Acad Sci USA 107:18138–18142. https://doi.org/10.1073/pnas.1010318107

Bogachev AV, Belevich NP, Bertsova YV, Verkhovsky MI (2009) Primary Steps of the Na^+^-translocating NADH:Ubiquinone Oxidoreductase Catalytic Cycle Resolved by the Ultrafast Freeze-Quench Approach. Journal of Biological Chemistry 284:5533–5538. https://doi.org/10.1074/jbc.M808984200

Bogachev AV, Bertsova YV, Barquera B, Verkhovsky MI (2001) Sodium-Dependent Steps in the Redox Reactions of the Na^+^ -Motive NADH:Quinone Oxidoreductase from *Vibrio harveyi*. Biochemistry 40:7318–7323. https://doi.org/10.1021/bi002545b

Bogachev AV, Bertsova YV, Ruuge EK, Wikström M, Verkhovsky MI (2002) Kinetics of the spectral changes during reduction of the Na ^+^ -motive NADH:quinone oxidoreductase from *Vibrio harveyi*. Biochimica et Biophysica Acta (BBA) - Bioenergetics 1556:113–120. https://doi.org/10.1016/S0005-2728(02)00342-0

Borshchevskiy V, Round E, Bertsova Y, Polovinkin V, Gushchin I, Ishchenko A, Kovalev K, Mishin A, Kachalova G, Popov A, Bogachev A, Gordeliy V (2015) Structural and Functional Investigation of Flavin Binding Center of the NqrC Subunit of Sodium-Translocating NADH:Quinone Oxidoreductase from *Vibrio harveyi*. PLoS ONE 10:e0118548. https://doi.org/10.1371/journal.pone.0118548

Deka RK, Brautigam CA, Liu WZ, Tomchick DR, Norgard MV (2016) Molecular insights into the enzymatic diversity of flavin‐trafficking protein (Ftp; formerly ApbE) in flavoprotein biogenesis in the bacterial periplasm. MicrobiologyOpen 5:21–38. https://doi.org/10.1002/mbo3.306

Hau J-L, Kaltwasser S, Muras V, Casutt MS, Vohl G, Claußen B, Steffen W, Leitner A, Bill E, Cutsail GE, DeBeer S, Vonck J, Steuber J, Fritz G (2023) Conformational coupling of redox-driven Na^+^-translocation in *Vibrio cholerae* NADH:quinone oxidoreductase. Nat Struct Mol Biol 30:1686–1694. https://doi.org/10.1038/s41594-023-01099-0

Imkamp F, Biegel E, Jayamani E, Buckel W, Müller V (2007) Dissection of the caffeate respiratory chain in the acetogen *Acetobacterium woodii*: Identification of an Rnf-Type NADH dehydrogenase as a potential coupling Site. J Bacteriol 189:8145–8153. https://doi.org/10.1128/JB.01017-07

Juárez O, Nilges MJ, Gillespie P, Cotton J, Barquera B (2008) Riboflavin Is an Active Redox Cofactor in the Na^+^-pumping NADH:Quinone Oxidoreductase (Na^+^-NQR) from *Vibrio cholerae*. Journal of Biological Chemistry 283:33162–33167. https://doi.org/10.1074/jbc.M806913200

Juárez O, Shea ME, Makhatadze GI, Barquera B (2011) The Role and Specificity of the Catalytic and Regulatory Cation-binding Sites of the Na^+^-pumping NADH:Quinone Oxidoreductase from *Vibrio cholerae*. Journal of Biological Chemistry 286:26383–26390. https://doi.org/10.1074/jbc.M111.257873

Kaminski JW, Vera L, Stegmann DP, Vering J, Eris D, Smith KML, Huang C-Y, Meier N, Steuber J, Wang M, Fritz G, Wojdyla JA, Sharpe ME (2022) Fast fragment- and compound-screening pipeline at the Swiss Light Source. Acta Crystallogr D Struct Biol 78:328–336. https://doi.org/10.1107/S2059798322000705

Kishikawa J, Ishikawa M, Masuya T, Murai M, Kitazumi Y, Butler NL, Kato T, Barquera B, Miyoshi H (2022) Cryo-EM structures of Na^+^-pumping NADH-ubiquinone oxidoreductase from *Vibrio cholerae*. Nat Commun 13:4082. https://doi.org/10.1038/s41467-022-31718-1

Kuhns M, Trifunović D, Huber H, Müller V (2020) The Rnf complex is a Na^+^ coupled respiratory enzyme in a fermenting bacterium, *Thermotoga maritima*. Commun Biol 3:431. https://doi.org/10.1038/s42003-020-01158-y

Kumar A, Roth J, Kim H, Saura P, Bohn S, Reif-Trauttmansdorff T, Schubert A, Kaila VRI, Schuller JM, Müller V (2025) Molecular principles of redox-coupled sodium pumping of the ancient Rnf machinery. Nat Commun 16:2302. https://doi.org/10.1038/s41467-025-57375-8

Meng EC, Goddard TD, Pettersen EF, Couch GS, Pearson ZJ, Morris JH, Ferrin TE (2023) UCSF ChimeraX : Tools for structure building and analysis. Protein Sci 32:e4792. https://doi.org/10.1002/pro.4792

Muras V, Claussen B, Karuppasamy M, Schaffitzel C, Steuber J (2014) Continuous fluorescence-based measurement of redox-driven sodium ion translocation. Analytical Biochemistry 459:53–55. https://doi.org/10.1016/j.ab.2014.05.012

Steuber J, Vohl G, Casutt MS, Vorburger T, Diederichs K, Fritz G (2014) Structure of the *V. cholerae* Na^+^-pumping NADH:quinone oxidoreductase. Nature 516:62–67. https://doi.org/10.1038/nature14003

Tuz K, Mezic KG, Xu T, Barquera B, Juárez O (2015) The Kinetic Reaction Mechanism of the *Vibrio cholerae* Sodium-dependent NADH Dehydrogenase. Journal of Biological Chemistry 290:20009–20021. https://doi.org/10.1074/jbc.M115.658773

Vitt S, Prinz S, Eisinger M, Ermler U, Buckel W (2022) Purification and structural characterization of the Na^+^-translocating ferredoxin: NAD^+^ reductase (Rnf) complex of *Clostridium* *tetanomorphum*. Nat Commun 13:6315. https://doi.org/10.1038/s41467-022-34007-z

Westphal L, Wiechmann A, Baker J, Minton NP, Müller V (2018) The Rnf Complex Is an Energy-Coupled Transhydrogenase Essential To Reversibly Link Cellular NADH and Ferredoxin Pools in the Acetogen *Acetobacterium* *woodii*. J Bacteriol 200. https://doi.org/10.1128/JB.00357-18

Zhang L, Einsle O (2024) Architecture of the RNF1 complex that drives biological nitrogen fixation. Nat Chem Biol 20:1078–1085. https://doi.org/10.1038/s41589-024-01641-1
